# The Pilin N-terminal Domain Maintains *Neisseria gonorrhoeae* Transformation Competence during Pilus Phase Variation

**DOI:** 10.1371/journal.pgen.1006069

**Published:** 2016-05-23

**Authors:** Kyle P. Obergfell, H. Steven Seifert

**Affiliations:** Department of Microbiology-Immunology, Northwestern University's Feinberg School of Medicine, Chicago, Illinois, United States of America; Max Planck Institute for Terrestrial Microbiology, GERMANY

## Abstract

The obligate human pathogen *Neisseria gonorrhoeae* is the sole aetiologic agent of the sexually transmitted infection, gonorrhea. Required for gonococcal infection, Type IV pili (Tfp) mediate many functions including adherence, twitching motility, defense against neutrophil killing, and natural transformation. Critical for immune escape, the gonococcal Tfp undergoes antigenic variation, a recombination event at the *pilE* locus that varies the surface exposed residues of the major pilus subunit PilE (pilin) in the pilus fiber. This programmed recombination system has the potential to produce thousands of pilin variants and can produce strains with unproductive pilin molecules that are completely unable to form Tfp. Saturating mutagenesis of the 3’ third of the *pilE* gene identified 68 unique single nucleotide mutations that each resulted in an underpiliated colony morphology. Notably, all isolates, including those with undetectable levels of pilin protein and no observable surface-exposed pili, retained an intermediate level of transformation competence not exhibited in Δ*pilE* strains. Site-directed, nonsense mutations revealed that only the first 38 amino acids of the mature pilin N-terminus (the N-terminal domain or Ntd) are required for transformation competence, and microscopy, ELISAs and pilus purification demonstrate that extended Tfp are not required for competence. Transformation in strains producing only the pilin Ntd has the same genetic determinants as wild-type transformation. The Ntd corresponds to the alternative product of S-pilin cleavage, a specific proteolysis unique to pathogenic *Neisseria*. Mutation of the S-pilin cleavage site demonstrated that S-pilin cleavage mediated release of the Ntd is required for competence when a strain produces unproductive pilin molecules that cannot assemble into a Tfp through mutation or antigenic variation. We conclude that S-pilin cleavage evolved as a mechanism to maintain competence in nonpiliated antigenic variants and suggest there are alternate forms of the Tfp assembly apparatus that mediate various functions including transformation.

## Author Summary

*Neisseria gonorrhoeae*, the bacterium responsible for the disease gonorrhea, is capable of undergoing natural DNA transformation, a critical mechanism by which bacteria can take up DNA from the environment. Uptake of foreign DNA can lead to adaptation to a changing environment and allow the spread of antibiotic resistance, a particularly relevant issue in *N*. *gonorrhoeae*. It has been proposed that the Type IV pilus directly mediates transformation, however, our data show that the extended pilus is not required for transformation. Only a portion (the Ntd) of the pilin protein is required to maintain transformation ability and this domain is released by a unique proteolysis event termed S-pilin cleavage. Release of the Ntd allows cells to maintain competence during pilin antigenic variation, a process vital for immune escape that frequently produces cells that cannot form Type IV pili. While these cells were previously thought to be incapable of DNA transformation, our data suggest that S-pilin cleavage evolved as a mechanism that allows DNA transformation and horizontal gene transfer to occur in these cells lacking pili. We propose that a structurally similar but distinct pseudopilus mediates transformation.

## Introduction

*Neisseria gonorrhoeae* is a Gram-negative, obligate human pathogen and the etiological agent of gonorrhea [1]. Each year, the bacterium causes an estimated 106 million new cases worldwide[2].The rise of resistance to all available antibiotics coupled with the lack of any viable vaccine candidates have led the Centers for Disease Control to classify *N*. *gonorrh*oea*e* as an urgent threat, underscoring the need for understanding the fundamental virulence mechanisms of this organism.

The process of DNA transformation in *N*. *gonorrhoeae* and the closely related pathogen *Neisseria meningitidis* has been well-studied with the majority of contributing factors having been identified. All *N*. *gonorrhoeae* strains are naturally competent for DNA transformation, and competence is not regulated as the organism is able to undergo transformation during all phases of growth, which contributes to the spread of antibiotic resistance [3,4]. Transformation in *Neisseria* is mediated by the Type IV pilus (Tfp) complex. The Tfp is a major virulence factor involved in cellular adherence, microcolony formation, resistance to neutrophil mediated killing, twitching motility, and transformation [5–10]. Similar to *Haemophilus influenza*e, *N*. *gonorrhoeae* preferentially takes up its own DNA that contains a 10 or 12 base DNA uptake sequence (DUS) (5’ ATGCCGTCTGAA 3’) [11,12]. The DUS occurs frequently within Neisserial genomes and significantly increases efficiency of DNA transformation compared to the same DNA lacking the sequence [13].

Tfp are several micron long, six nm wide, dynamic structures undergoing cycles of extension and retraction and exert one of the largest forces known for a biological machine [14,15]. It has been assumed that Tfp directly bind DNA and retraction of the Tfp mediates DNA uptake into the periplasm; however, there is a lack of direct data supporting this model, and transporting DNA through the secretin that already hosts a pilus is problematic [16]. What is known is that the major pilin subunit (PilE or pilin), along with many of the proteins of the Tfp complex such as the prepilin peptidase PilD are required for competence [10,17]. PilT, a traffic ATPase that mediates retraction of pili, is required for transformation but is not required for piliation [18]. The minor pilin ComP is responsible for recognition and binding of DNA and is responsible for DUS recognition [19]. Expression of ComP promotes transformation while expression of the minor pilin PilV decreases transformation efficiency by antagonizing ComP [20].

The pilin structure is characteristic of Tfp pilin with a 7 AA leader peptide that is cleaved by the PilD peptidase from the N-terminal hydrophobic α-helices that form the core of the pilus structure. The α-helices are connected to a variable C-terminal domain consisting of the αβ loop, a β sheet, and the D Region containing a critical disulfide bond (S1A Fig). The globular C-terminal head contains the residues that are surface exposed in the pilus fiber [21]. Some pilin variants mediate release of the C-terminal globular head in a soluble form called S-pilin that is the product of a specific proteolysis unique to the pathogenic *Neisseria* [22,23]. No role for S-pilin in pathogenesis has been conclusively established. Pilin variants that produce fewer pili tend to produce larger amounts of S-pilin, but the ratio of pilin to S-pilin is not directly correlated to the amount of elaborated pili [22,24].

Both eukaryotic and prokaryotic pathogens utilize an array of molecular tactics in order to avoid recognition by host immune systems. Diversity generation systems are one of the most widely used of these tactics to avoid immune detection. From the V(D)J recombination mediated generation of antigen receptors in vertebrate adaptive immune responses to phase variation of antigenic determinants in bacteria, many different diversity generation systems exist throughout nature that are vital to an organism’s survival [25,26]. Pathogen antigenic variation, or the modification of immunogenic surface molecules, forces the host to continuously alter its humoral immune response. Antigenic variation can provide a pathogen with the capability to persist within a host for an extended time or to continually re-infect core populations.

The pathogenic *Neisseria* mediate a high-frequency, DNA-based, homologous recombination process termed pilin antigenic variation, that varies the *pilE* coding sequence resulting in many different variant pilins [16]. During pilin antigenic variation, sequences from unexpressed silent pilin loci (*pilS)* donate variable coding sequences to the expression locus in a gene conversion event. The *pilE* gene and pilin protein can be divided into variable regions (semi-variable region (SV), hyper-variable loop (HV_L_), hyper-variable tail (HV_T_)) and conserved regions (N-terminal domain, *cys1*, *cys2*) (S1B Fig). The regions with variable amino acid residues correspond to the surface exposed regions of the mature pilus fiber and are thought to mediate immune escape and add to the ineffectiveness of Tfp-based vaccine candidates. In addition, the process of pilin antigenic variation can result in pilin molecules that are inefficiently assembled into pilus fibers or pilin phase variants that cannot form pili [27]. All gonococcal strains maintain the ability to phase vary, which may contribute to immune escape by preventing pilus expression, but can additionally mediate the detachment of the bacterium from epithelial surfaces and a switch from a sessile biofilm state to a free-living planktonic state. Due to the observation that *pilE* deletion strains are nonfunctional for pilus-dependent processes [7,28,29], it has been assumed that when pilus expression is disrupted by the pilin antigenic variation process that all pilus associated functions are also disrupted.

In this report, we show that *pilE* mutations that prevent pilus formation do not necessarily abolish transformation competence. Mutations that disrupt the N-terminal domain (Ntd) prevent transformation, while those past the S-pilin cleavage site retain transformation competence. Moreover, we show that S-pilin proteolysis is required to release the Ntd to mediate transformation when unproductive pilin monomers are produced by antigenic variation. Our data demonstrate that a proteolytic process maintains transformation competence even when pilus expression is disrupted by pilin antigenic variation. These findings suggest that the extended pilus fiber is not required for transformation and that maintaining continual transformation competence is extremely important for this strict human pathogen.

## Results

### Targeted genetic screen to identify C-terminal pilin residues required for piliation

To determine the amino acid residues critical for proper Tfp formation and function we conducted a random, saturating mutagenesis screen on the 3’ region of the *pilE* coding sequence encoding the *cys1*, HV_L_, *cys2*, and HV_T_ regions (S1B Fig). A pool of random mutant plasmids was constructed using a series of degenerate mega-primers to introduce all possible mutations into every nucleotide position in the 3’ region of the gene. The plasmid pool was used to transform *N*. *gonorrhoeae* selecting for recombination at the native *pilE* locus using a linked, downstream chloramphenicol resistance marker (Cm^R^) (S1B Fig). Transformants with underpiliated (P-) colony morphologies were isolated and the *pilE* coding region was sequenced to determine the causative mutation. Only mutants containing a single nucleotide change in the *pilE* coding region were analyzed further.

68 unique mutations were isolated that result in a P- colony morphology in the targeted 132 3’-base pairs of *pilE* (S1C Fig). Nine of these mutations created early stop codons, 13 changed either of the two absolutely conserved cysteine residues that form the critical disulfide bond, and the remaining 46 were missense mutations. This mutagenesis screen provides a comprehensive analysis of which amino acid residues in the carboxy-terminus of this highly variable protein in this strain of *N*. *gonorrhoeae* are essential for piliation. Residues in which a mutation produced a P- phenotype were concentrated in the highly conserved *cys1* and *cys2* regions of *pilE*. There were, however, multiple amino acid residues in the hypervariable loop that prevent piliation when mutated. No mutations were isolated in the hypervariable tail consisting of the five most C-terminal amino acids (S1C Fig).

### Phenotypic analysis of P- *pilE* mutants

**Fig 1:**
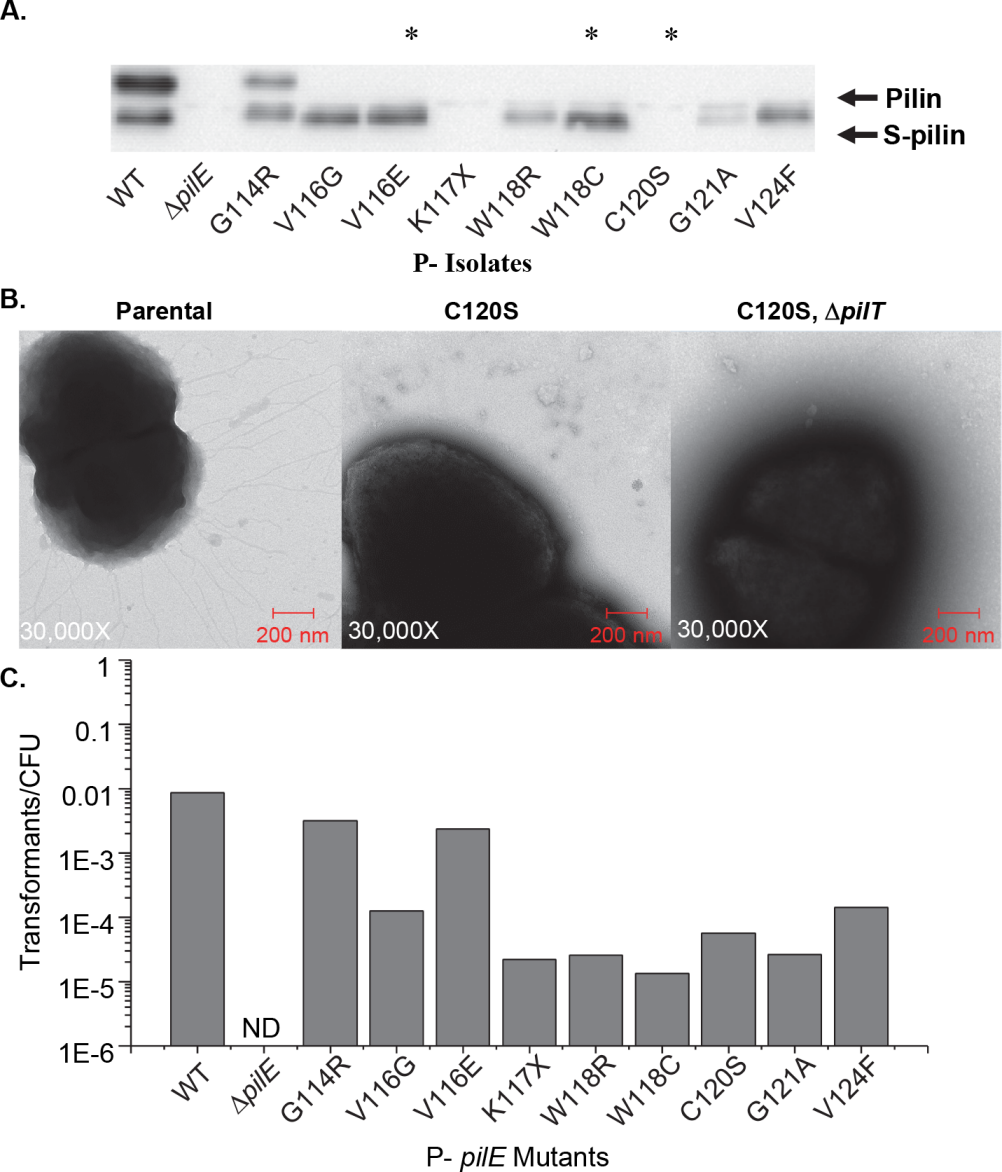
Phenotypic characterization of P- *pilE* mutants. **A.** Representative PilE western blot of whole cell lysates of P- isolates. The upper band is full-length PilE while the lower band is S-pilin. Lanes marked with an * are nonsense or cysteine mutants. Western blot analysis performed using the K36 peptide anti-pilin antibody. **B.** Representative TEM images of the parental strain (left), a C120S mutant (middle), and a C120S, *pilT* double mutant (right). **C.** Transformation efficiencies of representative P- isolates from the screen including nonsense, cysteine mutants, and missense mutants. X = nonsense mutation, ND = transformants not detected.

To further characterize representative mutant isolates for Tfp formation and function, pilin protein levels were assayed by western blot, surface exposed fibers were imaged by transmission electron microscopy (TEM), and transformation efficiencies were measured (Fig 1A–1C). While the missense mutants expressed varying levels of pilin, the nonsense and cysteine mutants had no detectable pilin by western blot analysis, presumably due to degradation of improperly folded pilin (Fig 1A). A complete summary of the phenotypic characterization of every isolate from the screen can be found in S1 Table. In addition, we were unable to detect any surface exposed pili by TEM with selected nonsense and cysteine mutants (Fig 1B). Several Tfp defects can be suppressed in a Δ*pilT* background, which prevents pilus retraction [30], yet a representative set of nonsense and cysteine mutants did not display any observable pili by TEM in a Δ*pilT* background (Fig 1B and S2 Fig). Surprisingly, while a Δ*pilE* mutant is not competent, all of the *pilE* mutants were transformable including the nonsense and cysteine mutants completely lacking detectable pilin or surface exposed pili (Fig 1C). The P- isolates demonstrated a wide range of transformation efficiencies, with mutants that express some detectable pilin protein on Western blot analysis generally exhibiting a higher level of transformation competence (S1 Table).

**Fig 2:**
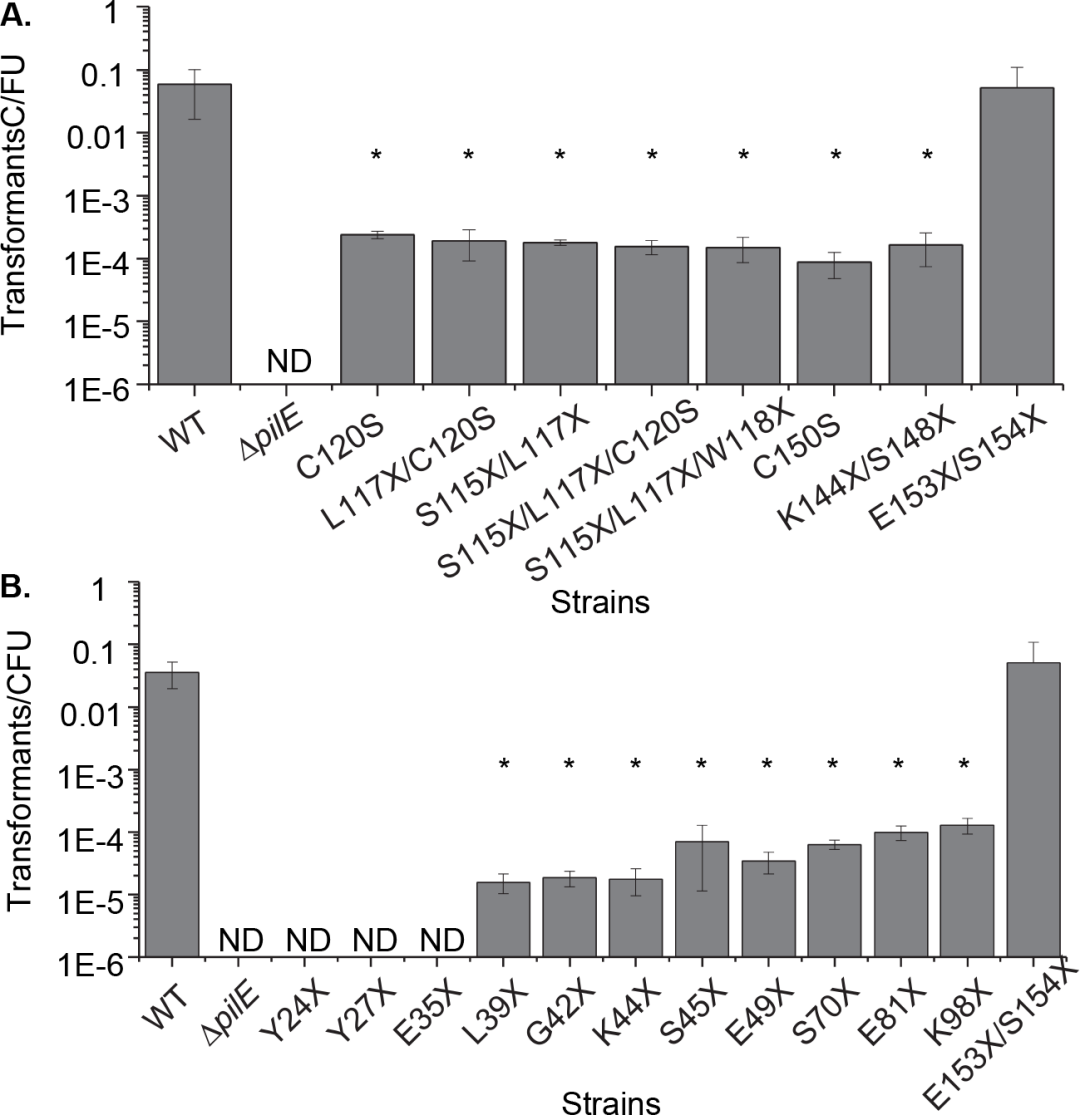
Nonsense mutants define the region of PilE necessary for transformation. **A.** Transformation efficiencies of strains with multiple site-directed nonsense and/or cysteine mutations of *pilE* designated by the original amino acid and the resultant amino acid or nonsense mutation (represented by the X). **B.** Transformation efficiencies of site-directed nonsense mutants of *pilE*. WT is the parental FA1090 strain and Δ*pilE* is a deletion derivative of that strain. X = nonsense mutation, ND = transformants not detected, *p<0.05 Student’s T-test, each mutant compared to the WT strain.

Although all experiments were performed in IPTG inducible RecA strain background to prevent antigenic variation, it was possible that the observed transformation was due to a low level of pilin antigenic variation restoring full-length pilin. We ruled out this possibility as all transformants obtained from the mutants retained their non-piliated colony morphology and sequencing of the *pilE* locus from a representative set of transformants showed no changes to the *pilE* sequence. We tested whether the competence of the P- isolates might be due to translational miscoding allowing a small amount of full-length pilin to be expressed from the mutants, since it has previously been shown that only a small amount of pilin expression is sufficient for competence [31,32]. If translational miscoding were responsible for the competence, insertion of consecutive nonsense mutations and cysteine mutations would lower transformation efficiency beyond that of a single mutant. However, upon introduction of two or three nonsense and cysteine mutants into the 3’ region of the gene, all mutant strains maintained a similar level of transformation as the individual nonsense and cysteine mutants isolated in the screen (Fig 2). Consistent with the lack of mutants isolated in the HV_T_ from the P- screen, insertion of consecutive nonsense mutations in the HV_T_ did not affect transformation efficiency (Fig 2). These data show that the full-length pilin protein is not required for a reduced but biologically relevant level of transformation competence.

### PilE Ntd-mediated transformation

Since a Δ*pilE* mutant was non-competent for transformation, but the *pilE* truncation mutants retained competence, we predicted that only the N-terminal portion of pilin is required to maintain competence. Nonsense mutations inserted along the length of the *pilE* coding region showed that strains with nonsense mutations after AA38 of mature pilin retained competence, while nonsense mutations prior to, or at AA34, abolished detectable transformation (Fig 2). For the remainder of the experiments in this report, we selected three representative non-piliated *pilE* mutations: K44X, a short nonsense mutant expressing only the N-terminal domain (Ntd); S115X/L117X/W118X, a triple C-terminal nonsense mutant; and C150S, a C-terminal cysteine mutant. All three of these mutants retain competence but completely lack pili as shown by pilus purification and electron microscopy, even when *pilT* was inactivated (S2 Fig). Additionally, while Tfp could be detected in a parental strain, no short pilus filaments, or alternate filaments formed by the Ntd, were detected in the Ntd expressing K44X strain by whole-cell enzyme-linked immunosorbent assay (ELISA) or immuno-gold TEM using a polyclonal anti-Ntd antibody (S3 Fig). Taken together, these results confirm that Tfp are not required for competence and only the pilin Ntd is required to allow transformation competence.

**Fig 3:**
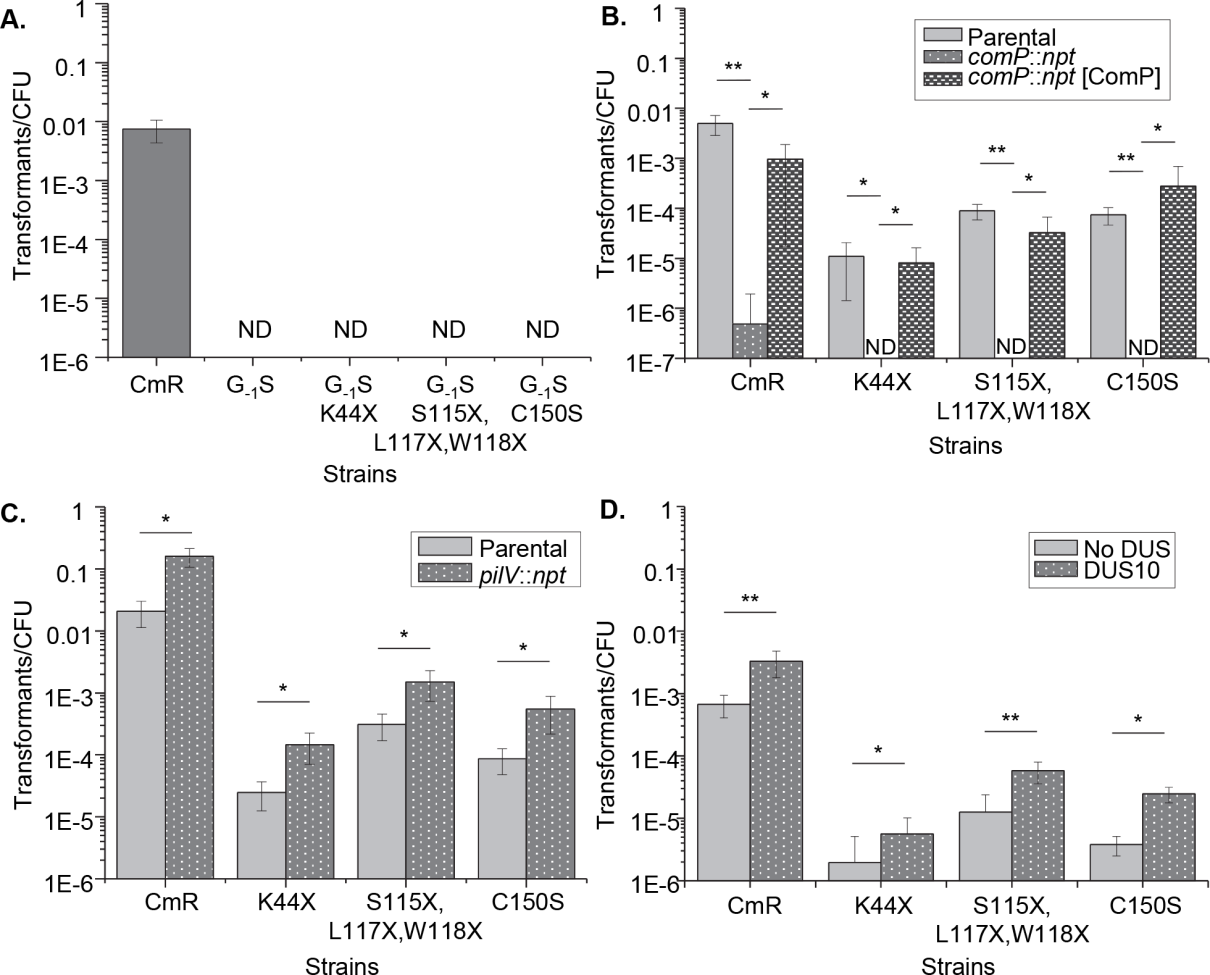
Pilin processing and role of minor pilins in Ntd-mediated transformation. **A.** Transformation efficiencies of strains with a *pilE* G_-1_S mutation that prevents PilD processing coupled with representative C-terminal nonsense mutations preventing piliation. **B.** Transformation efficiencies of *comP*::*npt* loss-of-function strains with *pilE* C-terminal nonsense mutations preventing piliation. [ComP] complements have an anhydrotetracycline (ATC) inducible copy of *comP* at an ectopic locus. **C.** Transformation efficiencies of *pilV***::***npt* strains with C-terminal *pilE* nonsense mutations preventing piliation. **D.** Transformation efficiencies of strains with C-terminal *pilE* nonsense mutations preventing piliation using as transforming DNA a plasmid either containing a 10-mer DUS (DUS10) or a scrambled DUS (No DUS). CmR = Cm^R^ parental strain, X = nonsense mutation, ND = transformants not detected, *p<0.05 **p<0.001 Student’s T-test.

To determine whether Ntd-mediated transformation was similar or distinct from the normal transformation process, we assayed for transformation efficiency of Ntd strains in several genetic backgrounds with established effects on transformation. The role of PilD was tested by introducing a G_-1_S mutation in PilE that prevents PilD cleavage [23] (Fig 3A). We also investigated the role of the DNA binding minor pilin, ComP (Fig 3B), and the competing minor pilin PilV (Fig 3C), by creating insertional mutants in the respective genes. The requirement for a DUS was assayed by using matched constructs for transformation that only differ in the presence or absence of a DUS (Fig 3D) [13]. Transformation in *pilE* mutants has the same genetic determinants as the normal process as all strains required PilD cleavage, ComP and a DUS for full transformation efficiency, while the PilV knockouts showed increased frequencies of transformation. The reliance on PilD cleavage also suggests that the Ntd must be present in the inner membrane or periplasm to mediate transformation as PilD cleavage occurs following transport of the pilin molecule through the inner membrane [33].

### S-pilin Cleavage and the Ntd

**Fig 4:**
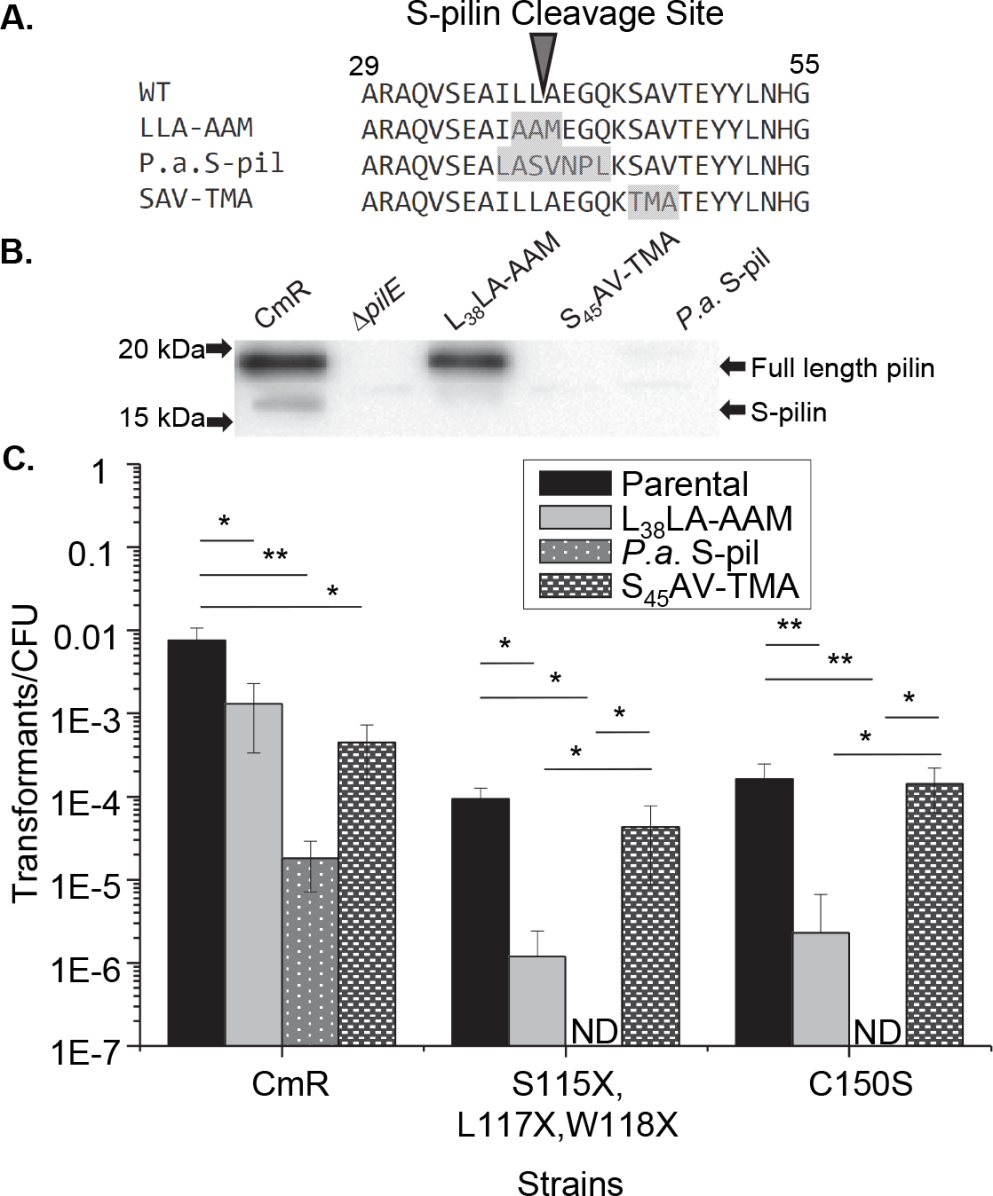
S-pilin cleavage is necessary for transformation competence in *pilE* mutant strains. **A.** Alignment of the PilE primary sequence surrounding the S-pilin cleavage site of the parental strain and the S-pilin cleavage mutant strains. **B.** PilE western blot of parental strain (CmR), Δ*pilE* mutant, *pilE* L_38_L_39_A_40_-AAM S-pilin cleavage mutant, a strain with *P*. *aeruginosa* PilA sequence at residues 37–43, and the S-pilin control mutation S_45_A_46_V_47_-TMA. Upper band is full-length pilin. Lower band is the processed S-pilin form. Western blot analysis performed using the K36 peptide anti-pilin antibody. **C.** Transformation efficiencies of a *pilE* C-terminal mutations coupled with the S-pilin cleavage site mutation (L_38_L_39_A_40_-AAM), *P*. *aeruginosa pilA* sequence (*P*.*a*. S-pil), or the control mutation (S_45_A_46_V_47_-TMA). X = nonsense mutation, CmR = Cm^R^ parental strain, *p<0.05 **p<0.001 Student’s T-test. ND = transformants not detected.

It was striking that the minimal N-terminal fragment necessary for transformation competence was 38 amino acids long, as S-pilin cleavage occurs between amino acids 39 and 40 of the mature pilin protein. We reasoned that the C-terminal PilE mutants unable to assemble pilus fibers were undergoing S-pilin cleavage to allow release of the Ntd to mediate transformation. Site-directed mutagenesis was used to make a set of double mutants in which a C-terminal mutation that abrogates piliation was coupled with a L_38_L_39_A_40_-AAM mutation at the S-pilin cleavage site that was previously described to prevent the majority of S-pilin cleavage, with the caveat that this mutation reduces overall piliation (Fig 4A) [34]. This previously reported phenotype in strain MS11 was confirmed in newly constructed L_38_L_39_A_40_-AAM mutants in strain FA1090 (Fig 4B). The C-terminal mutant strains each showed a drastic decrease in competence when the S-pilin cleavage site was mutated (Fig 4C). As expected, the L_38_L_39_A_40_-AAM mutation did not completely abrogate transformation as the mutation does not completely prevent S-pilin cleavage (Fig 4B).

In the same report describing the L_38_L_39_A_40_-AAM, Aas et al. also replaced the S-pilin cleavage region with the corresponding seven amino acid region from *Pseudomonas aeruginosa* pilin, PilA (Fig 4A)[34]. While this replacement mutation reduced transformation efficiency and destabilized Tfp in a WT background, it also prevented all production of S-pilin (Figs 4B and S4). Full-length pilin protein can be detected in this *P*. *aeruginosa* S-pilin (*P*.*a*. S-pil: I_37_LLAEGQ-LASVNPL) strain using a monoclonal antibody IE8G8 whose binding epitope is not altered by the *P*. *aeruginosa* sequence (S4A Fig). When we constructed this mutation in strains harboring a C-terminal *pilE* mutation, transformation was completely abolished (Fig 4C). The decrease in transformation efficiency observed with either of the S-pilin cleavage mutations was specific to mutation of the S-pilin cleavage site as a similar mutation (S_45_A_46_V_47_-TMA) just downstream of the S-pilin cleavage site did not decrease transformation efficiency when coupled with a C-terminal mutation. Moreover, this control mutation, in a WT *pilE* background, resulted in a P- colony morphology and completely destabilized full length pilin protein with only S-pilin being detected in concentrated cell supernatants(Fig 4B and 4C and S4 Fig). These data demonstrate that while any destabilizing mutation in the C-terminal region will reduce the transformation efficiency, when the S-pilin cleavage site is disrupted, the retained competence is lost.

Complementation of a Δ*pilE* strain was accomplished with a copy of *pilE* or the Ntd with an anhydrotetracycline (ATC) inducible promoter at an ectopic site. These constructs restored transformation efficiency in a Δ*pilE* background although not to parental levels (S5 Fig). The ability of the complementation constructs to express *pilE* transcripts relative to parental levels was measured by quantitative RT-PCR (S5A Fig). *pilE* transcript levels were reduced by about 1.5 logs in the complementation strains and the transformation efficiencies of the Δ*pilE iga*::*pilE* and Δ*pilE iga*::*pilE*_Ntd_ strains were reduced by a similar amount in comparison to the CmR and L39X strains respectively (S5 Fig). The transformability of the Δ*pilE iga*::*pilE*_Ntd_ strain demonstrates that the Ntd is sufficient to mediate transformation in a PilE deletion strain. This result, along with the transformation data from the S-pilin cleavage mutants and the demonstrated loss of competence of any nonsense mutation upstream of the S-pilin cleavage site proves that release of the Ntd by S-pilin cleavage is required to maintain transformation competence.

### The role of Ntd-mediated transformation during antigenic variation

**Fig 5:**
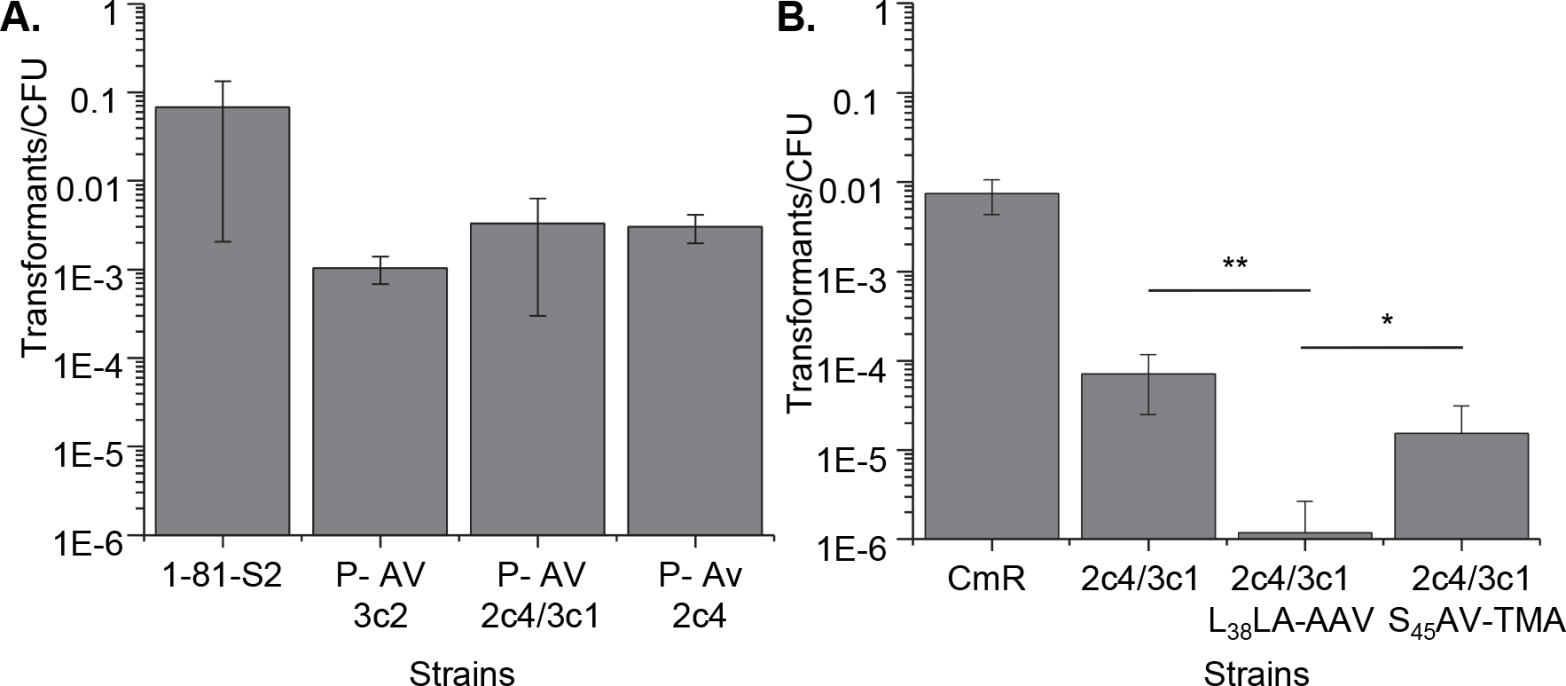
**Pilus phase variants require S-pilin cleavage for competence A.** Transformation efficiencies of naturally occurring P- antigenic variants with *pilE* variants that encode frameshift mutations resulting in early stop codons. **B.** Transformation efficiencies of P- strains resulting from antigenic variation events from donor silent loci 2c4 and 3c1 coupled with either the S-pilin cleavage mutation (L_38_L_39_A_40_-AAM) or the control mutation (S_45_A_46_V_47_-TMA). 3c2, 2c4/3c1, 2c4 indicate silent loci which donated sequence to the variant. CmR = Cm^R^ parental strain, *p<0.05 **p<0.001 Student’s T-test.

One of the many possible outcomes of pilin antigenic variation is phase variation or the creation of pilin molecules that cannot efficiently assemble into pilus fibers [35]. Some pilus phase variants are the outcome of frameshifts encoded in silent copies that result in early stop codons very similar to the nonsense mutations isolated in our screen, but with additional amino acid changes between the frame shift and the stop codon [27]. To determine whether naturally occurring pilus phase variants retained competence, we allowed the parental strain to undergo antigenic variation and isolated several unique *pilE* frameshift phase variants which resulted in nonsense mutations. We tested these naturally occurring nonpiliated variants for transformation efficiency, and each retained considerable competence (Fig 5A). Notably, the P- pilin antigenic variant strains exhibited higher transformation efficiencies than the strains with similar nonsense mutations created by site-directed mutagenesis. qRT-PCR of the *pilE* transcript showed that the Cm^R^ used to select the mutants actually decreased the *pilE* transcript to a third of wild-type levels resulting in lower PilE levels (S6A Fig). This decrease in mRNA corresponded with a decrease in transformation efficiencies as mutants isolated without a selection marker displayed greater competence than strains with the same mutation coupled to the Cm^R^ (S6B Fig).

Finally, we tested whether the L_38_L_39_A_40_-AAM mutation, when introduced into a natural P- phase variant also reduced transformation efficiency (Fig 5B). Consistent with the strains harboring other C-terminal mutations, the S-pilin cleavage mutation drastically reduced transformation efficiency of the phase variant, demonstrating that S-pilin cleavage is required for transformation in natural antigenic variants with unproductive pilin molecules. Taken together, these results strongly suggest that the Ntd is released by S-pilin proteolysis and can substitute for full-length pilin by producing a form of the pilus assembly apparatus that is active for DNA transformation during the normal process of pilus phase variation.

## Discussion

We have shown in a directed mutational analysis of the 3’ *pilE* coding region that a variety of *pilE* mutations that either alter or completely abolish pilus elaboration on the bacterial cell surface do not prevent transformation. A series of site-directed *pilE* mutants demonstrated that this transformation competence is not due to translational read-through, but that the maintenance of competence requires the pilin Ntd, a putative cleavage product of S-pilin proteolysis. Moreover, naturally occurring pilin variants that have pilin molecules incapable of forming an extended pilus fiber also retain competence. In these variants, S-pilin cleavage is required for transformation to mediate release of the Ntd from the mutated C-terminal head. We propose that the process of S-pilin cleavage releases the Ntd to maintain competence in cells undergoing antigenic variation, a diversity-generation system critical for immune escape during infection.

In comparing naturally occurring pilin phase variants to site-directed *pilE* mutants, we determined that our method for selecting *pilE* mutants with a Cm^R^ in the 3’ Sma/Cla region had the unintended consequence of suppressing *pilE* mRNA levels. We assume the Cm^R^ marker has interrupted an mRNA stability element, but further investigation will be required to define the mechanism that this insertion affects mRNA levels. It is important to note that most of the reported transformation efficiencies in this study are lowered by the action of the Cm^R^ cassette on *pilE* transcript. Without the Cm^R^ cassette, Ntd mediated transformation can still be remarkably efficient with more than one in a thousand cells being transformed (Fig 5B and S3B Fig). While this level of transformation is significantly lower than that of the fully piliated parental strain, it is still greater or equal to the competence efficiencies reported for the related bacterium *N*. *meningitidis* [36] and other naturally competence bacterial species [37,38]. Additionally, there was a notable drop in transformation efficiency as the site-directed truncations approached amino acid 38, which is either due to instability of the shorter peptide or loss of residues important for interaction with other factors. However, we cannot substantiate this conclusion as the extreme hydrophobic nature of the α1-N domain of the Ntd (residues 1 to 28) has prevented detection by western blot and HPLC [39] and multiple epitope tagged versions of the Ntd were not stable and prevented transformation competence. However, the requirement of at least a 38 amino acid long *pilE* gene product for competence demonstrates that the Ntd is stable enough to supply substantial biological function.

Although the L_38_L_39_A_40_-AAM pilin cleavage site mutant inhibits S-pilin cleavage but does not completely prevent cleavage, and the *Pseudomonas* S-pilin mutation abrogates S-pilin production but destabilizes full-length pilin; transformation assays with these two strains demonstrate that release of the Ntd is critical for maintenance of competence when unproductive pilin molecules with C-terminal mutations are expressed. While we cannot presently determine whether S-pilin cleavage is required for transformation with a fully functional pilin, the fact that the L_38_L_39_A_40_-AAM mutation did decrease transformation efficiency in the parental strain leaves open the possibility that S-pilin cleavage has a role regardless of the functional status of pilin. However, this reduction in competence may also be due to decreased ability of full-length, L_38_L_39_A_40_-AAM pilin to polymerize into pili [34]. Regardless of the mechanism, these data show that the proteolytic cleavage that releases S-pilin also releases the reciprocal Ntd product to maintain competence.

To our knowledge, the production of S-pilin and the Ntd has only been described for the pathogenic *Neisseria*, organisms also notable for producing many different piliation states through the process of antigenic variation, but not other Tfp pili producing bacteria. We propose that S-pilin/Ntd proteolysis evolved as a mechanism to release the Ntd to maintain competence in the face of pilus phase variation. These organisms undergo high frequency pilin variation that not only modulates the immune epitopes on the pilus fiber but also mediates phase variation, which can allow for detachment of phase variants from cell surfaces or biofilms.

The role of S-pilin/Ntd cleavage in compensating for the consequences of pilin antigenic variation adds another layer of adaptation to the already complex and highly evolved system. This complexity further underscores the importance of this diversity generation system to *N*. *gonorrhoeae* pathogenesis. Furthermore, because these organisms protect competence during antigenic variation and undergo horizontal gene transfer so frequently that there is an inability to establish clonal lineages [40], it appears that horizontal gene transfer is a vital function for these human specific organisms. It is not settled why these and other naturally-competent, human-restricted organisms require continual horizontal gene transfer, but our discovery that there is a specific mechanism to maintain competence in an easily reversible non-piliated state supports the notion that there is strong selection to maintain competence.

While S-pilin cleavage mediated release of the Ntd may be unique to *Neisseria*, a functional role for the N-terminal alpha helix apart from the entire pilin molecule has been previously demonstrated. Esquivel et al showed that the H-domain of the archaeal type IV pilin regulates motility in *Haloferax volcanii* [41]. Additionally, other studies in organisms such as *Thermus thermophilus* have demonstrated that mutations in multiple Tfp complex proteins separate the functions of Tfp and natural transformation [42,43]. Although it remains to be seen if the Ntd of pilins in other organisms can have functional roles outside the context of the entire pilin molecule, it is clear that the paradigm of Tfp-mediated transformation needs to be reexamined.

These reports, along with the data demonstrating that Tfp are not absolutely required for transformation competence, suggest that the prevailing model of extended Tfp-mediated DNA binding and uptake is not necessary. We propose that that there is a pseudopilus structure [21,44], distinct from the pilus, which helps mediates transformation. Similar to the pseudopilus present in Gram positive bacteria that spans the thick peptidoglycan layer, a gram-negative, competence pseudopilus would span from the anchor in the inner membrane, across the periplasmic space and through the outer membrane [21]. In the pilin mutants described here, the pilin Ntd could form the pseudopilus within the Tfp complex to present the competence minor pilin ComP near the cell surface but not produce a fiber that extends beyond the immediate cell surface. Formation of a functional fiber by the Ntd is plausible as the Ntd forms the core of the Type IV pilus structure, and extremely short type IV pilins can form Tfp such as the sixty-one amino acid version in *Geobacter sulfurreducens* [45,46]. Alternatively, the Ntd may not be included in the pseudopilus fiber, but rather serve as a structural or signaling component that is present in the inner membrane to allow for pseudopilus formation. Consistent with this hypothesis, several minor pilins (pilH-K) required for Tfp biogenesis are included in the pilus fibers at very low amounts possibly acting as an initiation complex that primes pilus assembly [39,47,48].

The composition and formation of the pseudopilus may be differentiated from a pilus fiber by the profile of minor pilins. The data showing that loss of PilV increases transformation in the Ntd producing mutants suggests that both PilV and ComP compete for access to the Tfp complex. Notably, neither of these two minor pilins is required for formation of the canonical Tfp. If this distinct transformation apparatus exists, it is possible that both full-length PilE and the PilE-Ntd could form the core of the pseudopilus as all phenotypes observed with Ntd mediated transformation in this study were consistent with transformation mediated by full-length pilin. The decrease in transformation efficiency exhibited by PilE nonsense mutations may indicate that full-length PilE more efficiently forms a pseudopilus, but this efficiency difference may be due to a lower protein stability in Ntd mutant strains. If full-length pilin can complete the pseudopilus, this model could account for transformation competence amongst all competent species that express Tfp. Whether the processed Ntd or the full-length pilin protein is required for promoting transformation in piliated cells, the data presented here clearly show that an extended pilus is not required for transformation. It is also possible that extended Tfp mediate transformation during favorable conditions but that the alternative pseudopilus structure only mediates transformation when Tfp cannot be formed. Though this study focuses on transformation, it stands to reason that the remarkable diversity of pilus-mediated functions coupled with a variety of minor pilins corresponding to different functions may allow formation of multiple alternate arrangements of the Tfp apparatus, each mediating a distinct process.

## Materials and Methods

### Bacterial strains and growth

All studies were performed using strain FA1090 PilE variant 1-81-S2 [49] and its derivatives which contain an IPTG inducible *recA6* allele to control pilin antigenic variation [50]. The Δ*pilE* mutant allele consists of a 924-bp deletion that includes the promoter and ribosome binding site of *pilE* as previously described [51]. The Δ*pilT* TEM experiments were performed in strains with an IPTG-regulatable *pilT* allele without IPTG induction [18]. *N*. *gonorrhoeae* strains were grown on GC Medium Base (Difco) plus Kellogg supplements I and II (GCB) at 37°C in 5% CO_2_. Antibiotics and their concentrations used for selection in GCB were: Chloramphenicol (Cm) 1 ug/ml, Kanamycin (Kan) 50 ug/ml, Naldixic Acid (Nal) 0.75 ug/ml, and Erythromycin (Erm) 2 ug/ml. Plasmids were propagated in One Shot TOP10 Electrocomp *E*. *coli* (Invitrogen) or E. cloni 10G ELITE Electrocompetent *E*. *coli* (Lucigen). *E*. *coli* strains were grown on Luria-Bertani (LB) solid media containing 15g/L agar or in broth at 37°C. Antibiotics and their concentrations used for selection in LB were: Kanamycin (Kan) 50 ug/ml, Ampcillin (Amp) 100 ug/ml, Chloramphenicol (Cm) 20 ug/ml, Tetracycline (Tet) 12 ug/ml.

### Construction of pKP11 and pKP37

PCR was used to amplify the *pilE* gene from strain FA1090 1-81-S2 *recA6*. KOD DNA polymerase (Novagen) was used following manufacturers protocols using kinase treated primers (T4 polynucleotide kinase, NEB) KP001 and KP002 for pKP11 and KP040 and KP002 for pKP37. Gel purified products (QIAquick Gel Extraction Kit, Qiagen) were cloned into pSMART LCAmp (Lucigen) following manufacturer’s instructions and electroporated in *E*. *coli* E.cloni 10g elite cells (Lucigen). Positive clones were confirmed by DNA sequencing using primers SL1 and SR2. Plasmids were isolated using the QIAprep Spin Miniprep Kit (Qiagen), digested with SmaI (NEB), and CIP treated (NEB). The Cm resistance cassette was PCR amplified with primers KP005 and KP006 to add a 12-mer DUS to the sequence and blunt cloned into the SmaI digested plasmids using T4 DNA ligase (NEB). The reaction was electroporated into *E*. *coli* Top10 cells and positive clones were confirmed by DNA sequencing.

### Single, degenerate, mega-primer mutagenesis

The pKP11 plasmid containing the *pilE* coding sequence was mutagenized by linear amplification using two synthetic 90-mer oligonucleotides (KP013 and KP014) with 12 base conserved flanking regions of homology and a central 66 base degenerate stretch (IDT) as megaprimers targeting the 3’ 132 coding nucleotides of *pilE*. The degenerate stretch was made by doping the synthesis reaction with 0.5% of each of the incorrect nucleotide resulting in the inclusion on average of one wrong base per oligonucleotide. The reactions were composed of KOD DNA polymerase 0.02 U/ul, MgSO_4_ 2.0 mM, dNTPs 0.2 mM each, pKP11 50 ng, and degenerate mega-primer 0.4 μM in 1X KOD reaction buffer. Following initial denaturation at 96°C for 2 min, linear amplification consisted of 18 cycles of 96°C for 1 min, 55°C for 1 min and 68°C for 8 min. Reactions were purified using the QIAquick PCR Purification Kit (Qiagen) and the template DNA was digested using 30U DpnI (NEB) overnight at 37°C. Reactions were dialyzed using 0.025 μm VSWP membrane discs (Millipore) and electroporated into *E*. *coli* E.cloni cells. Positive clones were selected on LB plates containing Amp and Cm, and mutant pools of several hundred were isolated by Miniprep reactions (Qiagen). DNA sequencing of select clones confirmed that ~10% of isolates contained a single nucleotide mutation in the region of interest. Mutant plasmid pools were used to transform FA1090 1-81-S2 *recA6*, and Cm^R^, P- transformants were isolated.

### Construction of *pilE* mutants

All site-directed PilE mutants were made through single-primer mutagenesis of pKP11 and pKP37 depending on the location of the desired mutation. To mutate the plasmid, a linear amplification step was carried out with primers (KP016,021,023,024,026,043–051,054–056,155,159,162, or 227) homologous to the region of interest with the nucleotide change required to mutate the desired site. The primer was used in a linear amplification step with subsequent processing and electroporation into *E*. *coli* in the same reaction manner as described for the degenerate mega-primer mutagenesis. Selection of correct clones was accomplished through PCR amplification of the *pilE* sequence of Cm^R^ isolates using primers KP001 and KP010 for pKP11 and KP040 and KP010 for pKP37 and subsequent DNA sequencing to select those plasmids incorporating the desired mutation. Double and triple mutants were constructed by repeating this process using additional mutagenic primer(s).

Markerless PilE mutant strains were made by PCR amplifying the *pilE* coding region of strains containing the desired mutation using primers KP173 and KP174, which contain a DUS but do not amplify the region with the inserted Cm^R^ originally used to make the mutation. The PCR reaction was performed using KOD polymerase following manufacturer’s conditions and purified using the QIAquick PCR Purification Kit. The resulting DNA was used to spot transform FA1090 1-81-S2 RecA6 and possible transformants were screened using visual assays for the P- colony morphology. All P- colonies were isolated and selection of correct clones whose P- phenotype was due to harboring the desired *pilE* mutation was accomplished through PCR amplification of the *pilE* and subsequent DNA. Complementation of the Δ*pilE* strain was accomplished by inserting a copy of *pilE*, amplified from the parental strain (*iga*::*pilE)* or the markerless L39X strain (*iga*::*pilE*_Ntd_) using primers KP221 and KP222, under an anhydrotetracycline inducible promoter at the *iga* protease locus using plasmid pMR69 [52].

### Construction of *comP*::*npt* and *pilV*::*npt*

*comP* and *pilV* sequences were amplified from strain FA1090 using kinase treated primers KP176 and KP177 (*comP)*, and KP178 and KP179 (*pilV*) using KOD DNA polymerase. Gel purified products were cloned into pSMART LCAmp and electroporated into *E*. *coli* BH10B cells. Positive clones were confirmed by DNA sequencing and the labeled pSM9 for the *pilV* construct and pSM18 for the *comP* construct. For the *pilV* construct pSM9, site directed mutagenesis was carried out using primers KP180 and KP181 to insert KpnI digestion sites. The resultant construct was named pSM12. pSM12 and pSM18 were digested by KpnI and PstI respectively and both were ligated using T4 DNA ligase with the nptII cassette digested from pBSL86 to insert the Kan^R^ gene into the coding sequence of *pilV* and *comP*. The resulting plasmids were electroporated into *E*. *coli* BH10B cells and positive clones were confirmed by DNA sequencing. The sequencing confirmed constructs pSM14 (*pilV*::*npt*) and pSM19 (*comP*::*npt*) were transformed into their native loci in FA1090 1-81-S2 *recA6* and its derivatives, selected for Kan^R^ and confirmed by DNA sequencing. Complementation of the *comP*::*npt* strain was accomplished by inserting a copy of *comP*, amplified using primers KP157 and KP158, under an anhydrous tetracycline inducible promoter at the *iga* protease locus using plasmid pMR69 [52].

### Transformation assays

*N*. *gonorrhoeae* strains were grown for 20 hours on GCB plates and resuspended in liquid transformation media (GCBL, 1mM IPTG, 5mM MgSO_4_ and Kellogg supplements I and II, pH 7.2) at high density. 20 μl of the cell suspension was added to 200μl transformation media containing 150ng pSY6 DNA [53]. For DUS experiments, 150 ng of either *gyrB1*DUS0 or *gyrB1*DUS10 [54], were used as the transforming DNA. After 20 min incubation at 37°C, the transformation reactions were diluted into 2ml 37°C transformation media and incubated at 37°C in the presence of 5% CO_2_ for 4 h. Reactions were then serially diluted and spotted onto GCB plates in the presence and absence of Nal. Transformation efficiencies are reported as antibiotic resistant CFU (transformants) divided by total CFU, and are the mean of at least three replicates.

### Western blots

Protein isolation from cell lysates was accomplished after growth of strains on GCB plates for 18 hours. Cells were swabbed into 1 mL PBS and pelleted at 4,000 x g for 5 minutes and washed with 500 ul PBS. Bacteria were resuspended in PBS to 520ul total volume. 20 μl was reserved for BCA analysis (Pierce) to determine protein concentration and 5x SDS sample buffer was added to the remaining 500 μl. To aid in loading of the sample, genomic DNA was sheared through repeated passage of the sample through a small bore needle and stored at -20°C. For western blots of concentrated pilin protein from cell supernatants (S4B Fig), strains were grown as lawns on GCB for 8 hours prior to inoculation of 5 ml of amended GCBL. (GCBL + 0.042% sodium bicarbonate). Following overnight growth at 30°C with rotation, cells were pelleted by ultracentrifugation at 200,000 x g for 1 hr at 4°C. Pilin protein in the supernatants was concentrated using trichloroacetic acid as described [22] and suspended in PBS and 5x SDS sample buffer. For western blot analysis equal amounts of protein were loaded onto 15% SDS-PAGE gels and run at 150 V using standard technique. Gels were blotted using CAPS buffer [10 mM 3-(cyclohexylamino)-1-propanesulfonic acid (pH 11.0); 10% methanol] to 0.45 μm polyvinylidene difluoride (PVDF) membrane using a Bio-Rad transfer cell at 100 v for 1 h at 4°C. Antibodies were used at the following dilutions: K36 (anti-PilE peptide) 1:50,000, IE8G8 (anti-PilE monocolonal Ab) 1:1,000 and 1:500, Peroxidase-conjugated AffinPure Gt α-Rabbit IgG (Jackson ImmunoResearch) 1:10,000. Western blots were developed using the Enhanced Chemiluminescence (ECL) Kit (GE Healthcare) following manufacturer’s instructions.

### Pilus filament purification

Purification of Tfp was performed based on previously described methods [18]. Bacteria were grown as lawns on GCB plates for 20 hours. Bacteria from 30 plates were suspended in 20 ml of 0.15 M ethanolamine pH 10.5 and pili were sheered for 30 seconds in a blender at high speed. Bacterial cells were pelleted by centrifugation at 17,000g, 4°C for 15 minutes. The supernatant containing the pilus filaments was precipitated with one tenth volume of ammonium sulfate saturated 0.15 M ethanolamine on ice for 30 minutes. Pili were pelleted by centrifugation at 17,000g, 4°C for 15 minutes. Supernatants were discarded and pellets were twice washed in 10 ml 0.05 M Tris buffered saline followed by centrifugation at 17,000g, 4°C for 15 minutes. Pili were solubilized in 100 μl PBS.

### Transmission electron microscopy

Strains were plated on GCB solid media for isolated colonies and grown for 18 hrs. 300-mesh nickel grids with carbon support films (Ladd Research) were touched to medium density colonies to pick up bacterial cells, fixed for 10 min in PBS, 4% PFA and 0.2% gluteraldehyde; washed 5X in sterile water for 5 min each; and negatively stained with 1–3% uranyl acetate for at least 1 min prior to imaging. Immuno-gold labeled samples were fixed for 10 min in PBS, 4% PFA and 0.2% gluteraldehyde; washed 3X in PBS, 1% bovine serum albumin (BSA); blocked for 30 min in PBS, 5% BSA and 0.1% gelatin; incubated with rabbit polyclonal anti-Ntd antibody (1:500 dilution); washed 3X in PBS, 1% BSA; blocked for 30 min in PBS, 5% BSA and 0.1% gelatin; incubated with 12nm Colloidal Gold-AffiniPure Goat Anti-Rabbit IgG (Jackson ImmunoResearch) (1:50 dilution); washed 5X in sterile water for 5 min each; and negatively stained with 1% uranyl acetate for at least 1 min prior to imaging. All imaging was done on a FEI Tecnai Spirit G2 120-kV at Northwestern’s Center for Advanced Microscopy.

### ELISA

Whole-cell ELISAs were carried out as described [55] with slight modifications. Strains were grown on GCB solid media as lawns for 20 hrs. Cells were swabbed into four ml PBS and diluted to an OD_550_ of 0.2. 2X serial dilution were used to inoculate a 96-well flat bottomed plate (Sarstedt) with 100 ul of culture per well with six repeats per condition. The plate was spun down at 3,220 x g for 10 min and the 75 ul of supernatant was removed. The remaining liquid was allowed to dry at 50°C until all liquid evaporated and the cells were fixed for 10 min at RT in 100 ul PBS, 4% PFA. The wells were washed 3X with PBS, and blocked for 10 min in 1% BSA in PBS 0.1% tween. Blocking solution was removed and 50 ul rabbit polyclonal anti-Ntd antibody (1:4,000 dilution in blocking solution) was added for 1 hr. Primary antibody was removed and wells were washed 3X with PBS and 50 ul peroxidase-conjugated AffinPure Gt α-Rabbit IgG (Jackson ImmunoResearch) (1:1,000 dilution in blocking solution) was added for 1 hr. Secondary antibody was removed and the wells were washed 3X in PBS. Assay was developed using 100 ul 3,3',5,5'-tetramethylbenzidine (TMB) substrate (10mg/ml TMB in DMSO diluted 1:100 in .01% hydrogen peroxide, citrate acetate buffer pH 6.0,) for 10 minutes and stopped by addition of 25 ul of 2M sulfuric acid as stop solution. Absorbance was measured 450nm with corrective absorbance at 570nm. Results are the average absorbance of four serial dilutions and three independent experiments.

### qRT-PCR

*pilE* transcript levels were determined by quantitative RT-PCR in strains grown in liquid culture, and total RNA was isolated and cDNA was amplified as previously described [56]. Relative transcript abundance of *pilE* was determined using the comparative Ct method [57] with the *omp3* transcript serving as the internal control using primers KP170 and KP171 (*pilE*), and KP182 and KP183 (*omp3*).

## Supporting Information

S1 Fig Single nucleotide mutations in *pilE* that result in a P- phenotype. **A.** Picture of the PilE pilin structure Protein Data Bank accession no. 2HI2 [45]. **B.** Gene map of *pilE* showing the regions of sequence conservation (grey) and variation (white) with the screened region boxed in light red. Triangle labeled Cm^R^ indicates location of linked chloramphenicol resistance cassette used to select for transformants. **C.** Graph depicting the location of mutations isolated in the screen that result in a P- phenotype. The x-axis depicts the amino acid residues corresponding to the *pilE* sequence with each residue divided into the 3 segments representing the coding nucleotides. Amino acids in red lettering resulted in a P- phenotype when mutated. The y-axis depicts the number of times a mutated residue was isolated.
(TIF)

S2 Fig Characterization of Piliation of PilE mutants. **A.** PilE western blot of pilus filament purification using monoclonal anti-PilE MAb IE8G8 at a 1:1,000 dilution. Pili were purified from the parental strain (CmR), Δ*pilE*, and *pilE* mutants in both a WT and Δ*pilT* strain background using an equal number of bacteria per strain. **B.** Representative electron micrographs of indicated strains in both a WT and Δ*pilT* strain background. X = nonsense mutation, CmR = Cm^R^ parental strain with 1-81-S2 *pilE* variant.
(TIF)

S3 Fig Analysis of Piliation in Ntd expressing strain. **A.** Quantification of pilus filament formation of strains using whole cell ELISA with a polyclonal, anti-Ntd antibody at 1:4,000 dilution. The Abs_450_ is plotted relative to the signal in the WT strain. n.s.–not significant indicated by Student’s T-test calculated p value above 0.05. **B.** Representative Immuno-gold TEM images of negatively stained *N*. *gonorrhoeae* strains with labeling of pilus filaments using a polyclonal, anti-Ntd antibody at 1:500 dilution. X = nonsense mutation.
(TIF)

S4 Fig **Pilin production by S-pilin mutant strains:** PilE western blots of whole cell lysates (**A.**) and concentrated cell supernatants (**B.**) of parental strain (CmR), Δ*pilE* mutant, *pilE* L_38_L_39_A_40_-AAM S-pilin cleavage mutant, a strain with *P*. *aeruginosa* PilA sequence at residues 37–43, and the S-pilin control mutation S_45_A_46_V_47_-TMA. Upper band is full-length pilin. Lower band is the processed S-pilin form. Western blot analysis performed using monoclonal anti-PilE MAb IE8G8 at a 1:500 dilution.
(TIF)

S5 Fig Complementation of ΔpilE strain with the Ntd. **A.** Relative mRNA levels of *pilE* in the presence or absence of ATC as measured by quantitative RT-PCR. **B.** Transformation efficiencies of Δ*pilE* complementation strains in the presence of ATC. Strains Δ*pilE iga*::*pilE* and Δ*pilE iga*::*pilE*_Ntd_ have an ATC inducible copy of *pilE* or the Ntd (PilE L39X) respectively inserted at the *iga* locus. X = nonsense mutation, CmR = Cm^R^ parental strain, -DNA = no transforming DNA added to reaction, *p<0.05, **p<0.001 Student’s T-test.
(TIF)

S6 Fig Chloramphenicol resistance cassette inserted downstream of *pilE* decreases Pilin expression. **A.** Relative mRNA levels of *pilE* as measured by quantitative RT-PCR. Strains CmR, E35X, K44X, K98X, and C150S contain a Cm^R^. **B.** Transformation efficiencies of strains either with or without the Cm^R^ downstream of *pilE*. X = nonsense mutation, ND = transformants not detected, *p<0.05 Student’s T-test.
(TIF)

S1 Table Phenotypic characterization of P- isolates. ^a^location of mutation in *pilE* resulting in a P- colony morphology. ^b^resultant amino acid change. Red shading indicates a nonsense mutation. Yellow shading indicates a cysteine mutant. ^c^Transformation competence relative to the WT strain (++++) and the non-competent Δ*pilE* strain. ^d^Pilin protein levels relative to the WT strain (++) and the Δ*pilE* strain (-). ^e^S-pilin protein levels relative to the WT strain (++) and the Δ*pilE* strain (-). ^f^Pilus related colony morphology relative to WT (++) and the Δ*pilE* strain (—).
(DOCX)

S2 Table Primers used in this study. (DOCX)
