# Supplementary figures and images for: The Pilin N-terminal Domain Maintains Neisseria gonorrhoeae Transformation Competence during Pilus Phase Variation
Source: PLoS Genet. 2016 May 23;12(5):e1006069. doi: 10.1371/journal.pgen.1006069 (PMC4877100; doi:10.1371/journal.pgen.1006069)

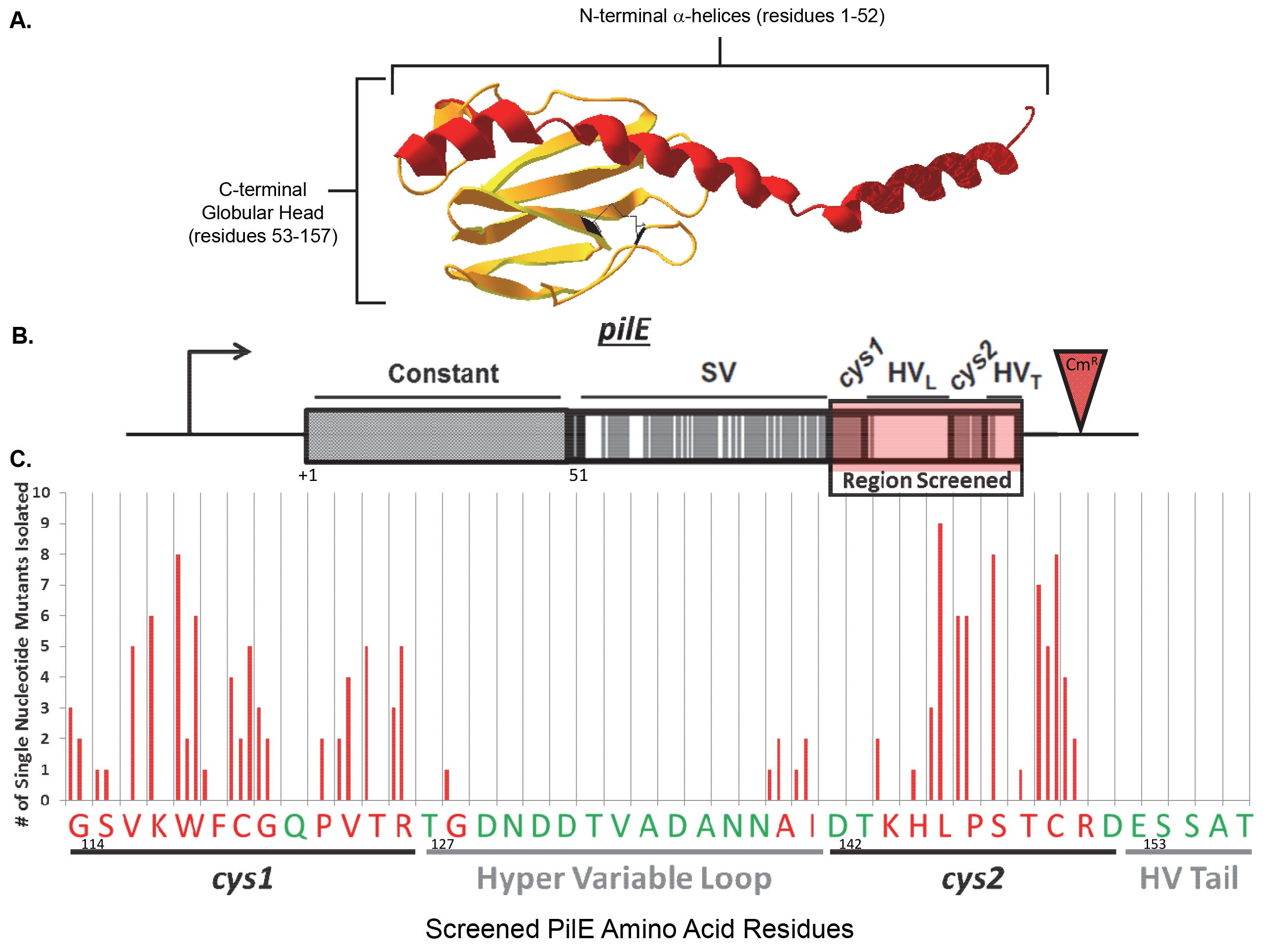

Supplement: S1 Fig — A. Picture of the PilE pilin structure Protein Data Bank accession no. 2HI2 [45]. B. Gene map of pilE showing the regions of sequence conservation (grey) and variation (white) with the screened region boxed in light red. Triangle labeled CmR indicates location of linked chloramphenicol resistance cassette used to select for transformants. C. Graph depicting the location of mutations isolated in the screen that result in a P- phenotype. The x-axis depicts the amino acid residues corresponding to the pilE sequence with each residue divided into the 3 segments representing the coding nucleotides. Amino acids in red lettering resulted in a P- phenotype when mutated. The y-axis depicts the number of times a mutated residue was isolated. (TIF) [file pgen.1006069.s001.tif]

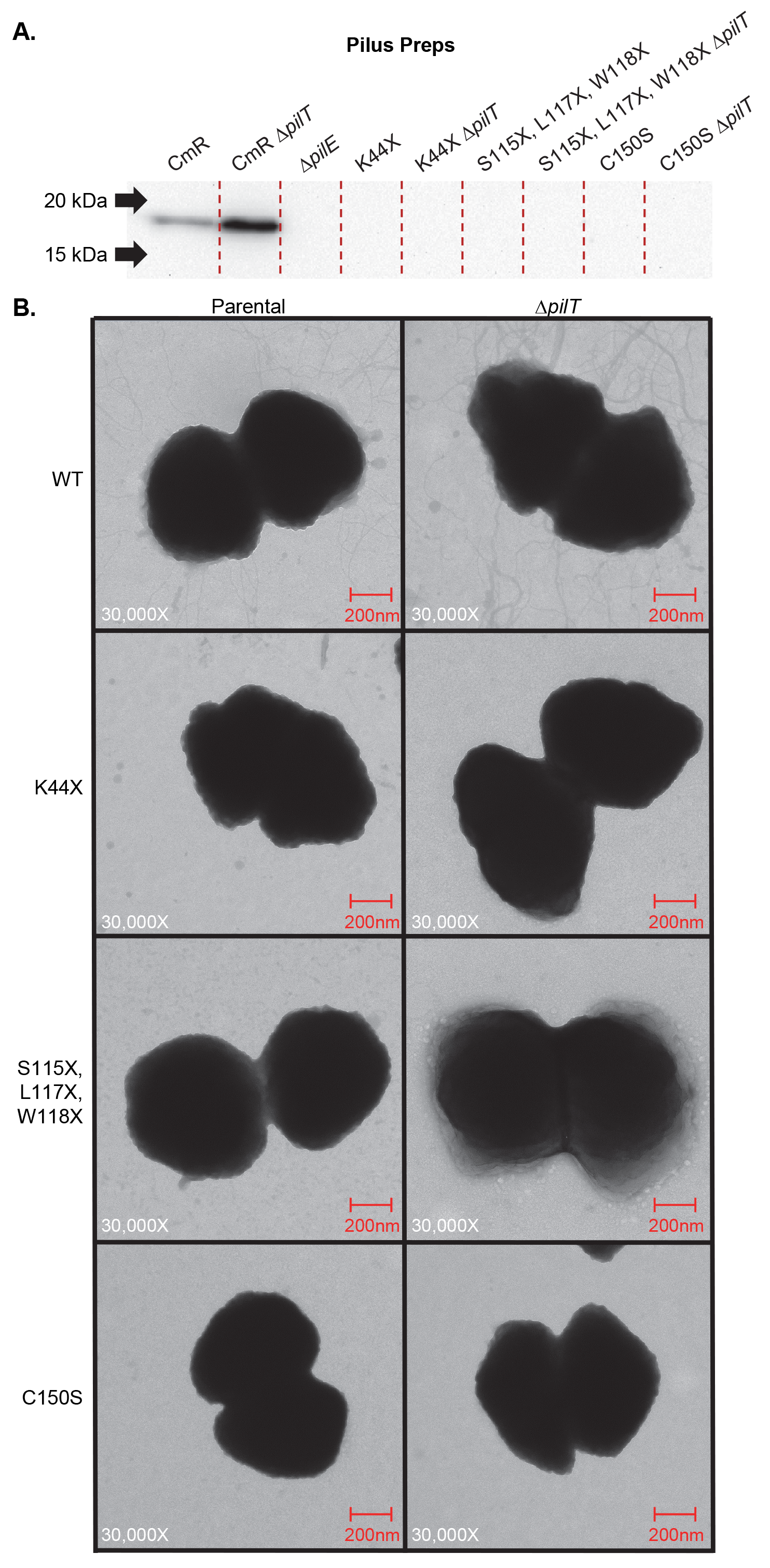

Supplement: S2 Fig — A. PilE western blot of pilus filament purification using monoclonal anti-PilE MAb IE8G8 at a 1:1,000 dilution. Pili were purified from the parental strain (CmR), ΔpilE, and pilE mutants in both a WT and ΔpilT strain background using an equal number of bacteria per strain. B. Representative electron micrographs of indicated strains in both a WT and ΔpilT strain background. X = nonsense mutation, CmR = CmR parental strain with 1-81-S2 pilE variant. (TIF) [file pgen.1006069.s002.tif]

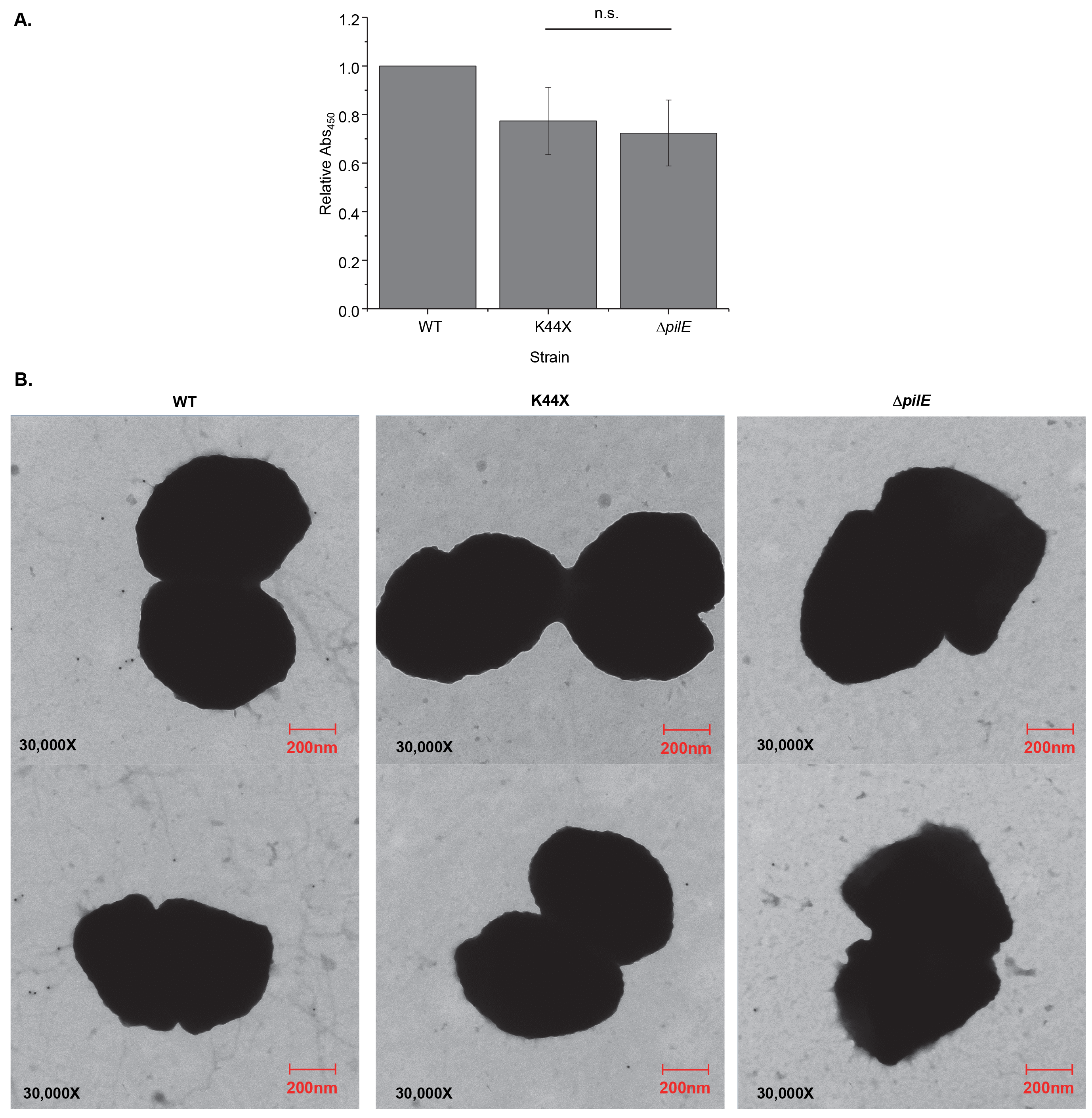

Supplement: S3 Fig — A. Quantification of pilus filament formation of strains using whole cell ELISA with a polyclonal, anti-Ntd antibody at 1:4,000 dilution. The Abs450 is plotted relative to the signal in the WT strain. n.s.–not significant indicated by Student’s T-test calculated p value above 0.05. B. Representative Immuno-gold TEM images of negatively stained N. gonorrhoeae strains with labeling of pilus filaments using a polyclonal, anti-Ntd antibody at 1:500 dilution. X = nonsense mutation. (TIF) [file pgen.1006069.s003.tif]

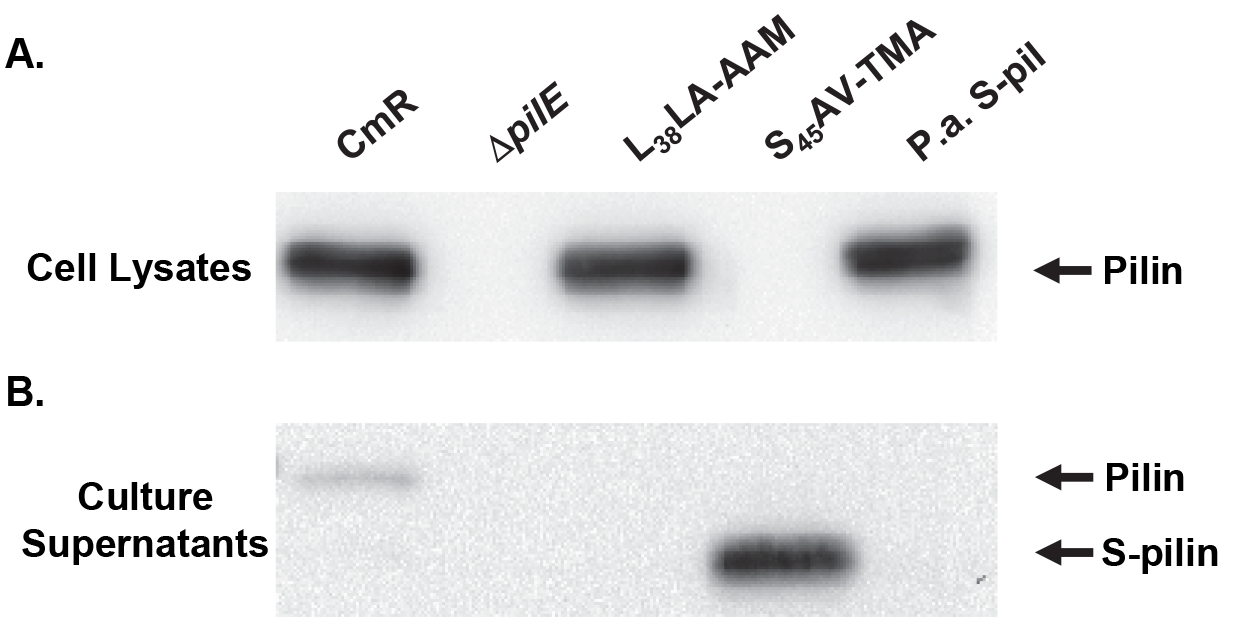

Supplement: S4 Fig — Pilin production by S-pilin mutant strains: PilE western blots of whole cell lysates (A.) and concentrated cell supernatants (B.) of parental strain (CmR), ΔpilE mutant, pilE L38L39A40-AAM S-pilin cleavage mutant, a strain with P. aeruginosa PilA sequence at residues 37–43, and the S-pilin control mutation S45A46V47-TMA. Upper band is full-length pilin. Lower band is the processed S-pilin form. Western blot analysis performed using monoclonal anti-PilE MAb IE8G8 at a 1:500 dilution. (TIF) [file pgen.1006069.s004.tif]

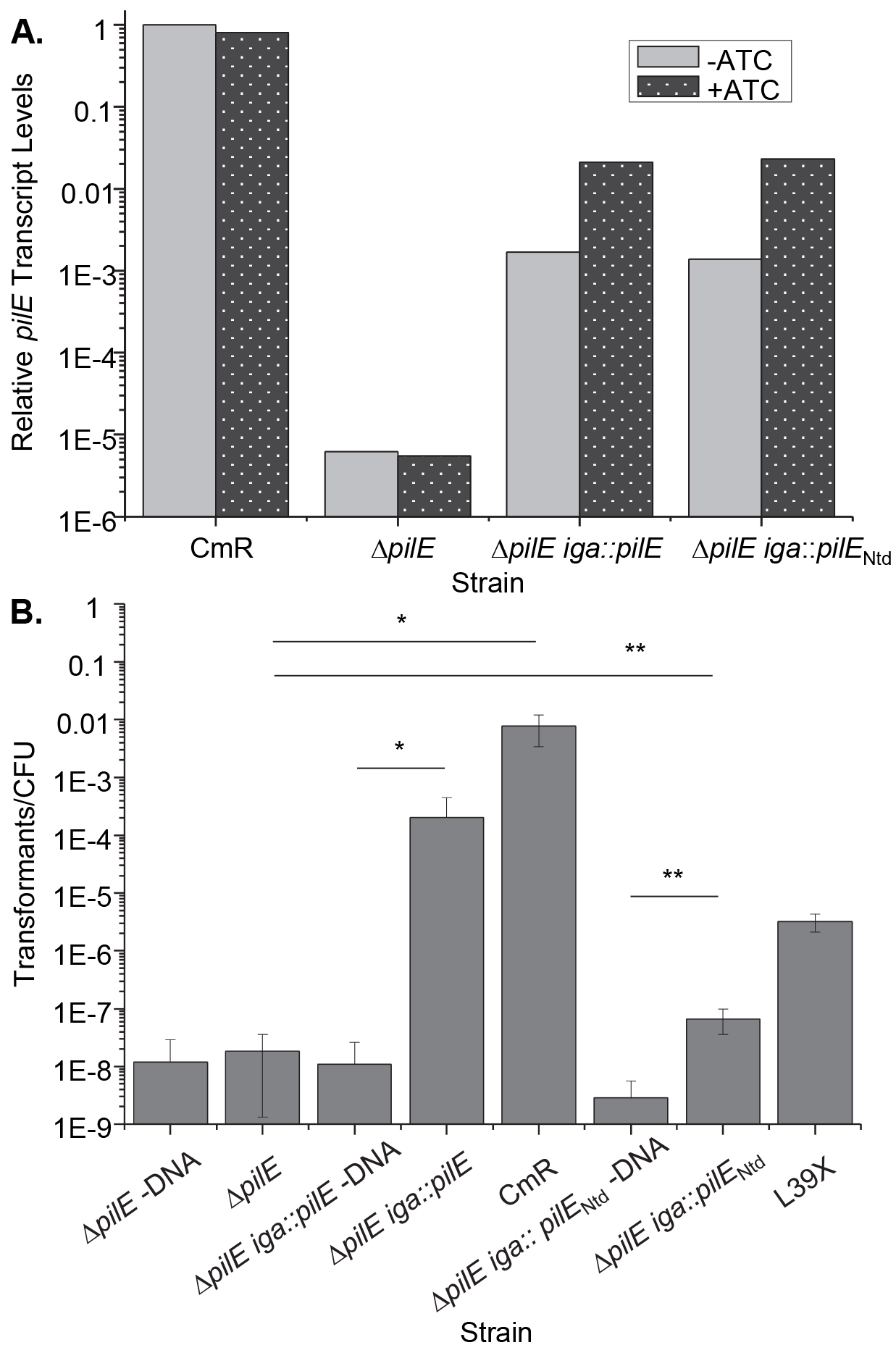

Supplement: S5 Fig — A. Relative mRNA levels of pilE in the presence or absence of ATC as measured by quantitative RT-PCR. B. Transformation efficiencies of ΔpilE complementation strains in the presence of ATC. Strains ΔpilE iga::pilE and ΔpilE iga::pilENtd have an ATC inducible copy of pilE or the Ntd (PilE L39X) respectively inserted at the iga locus. X = nonsense mutation, CmR = CmR parental strain, -DNA = no transforming DNA added to reaction, *p<0.05, **p<0.001 Student’s T-test. (TIF) [file pgen.1006069.s005.tif]

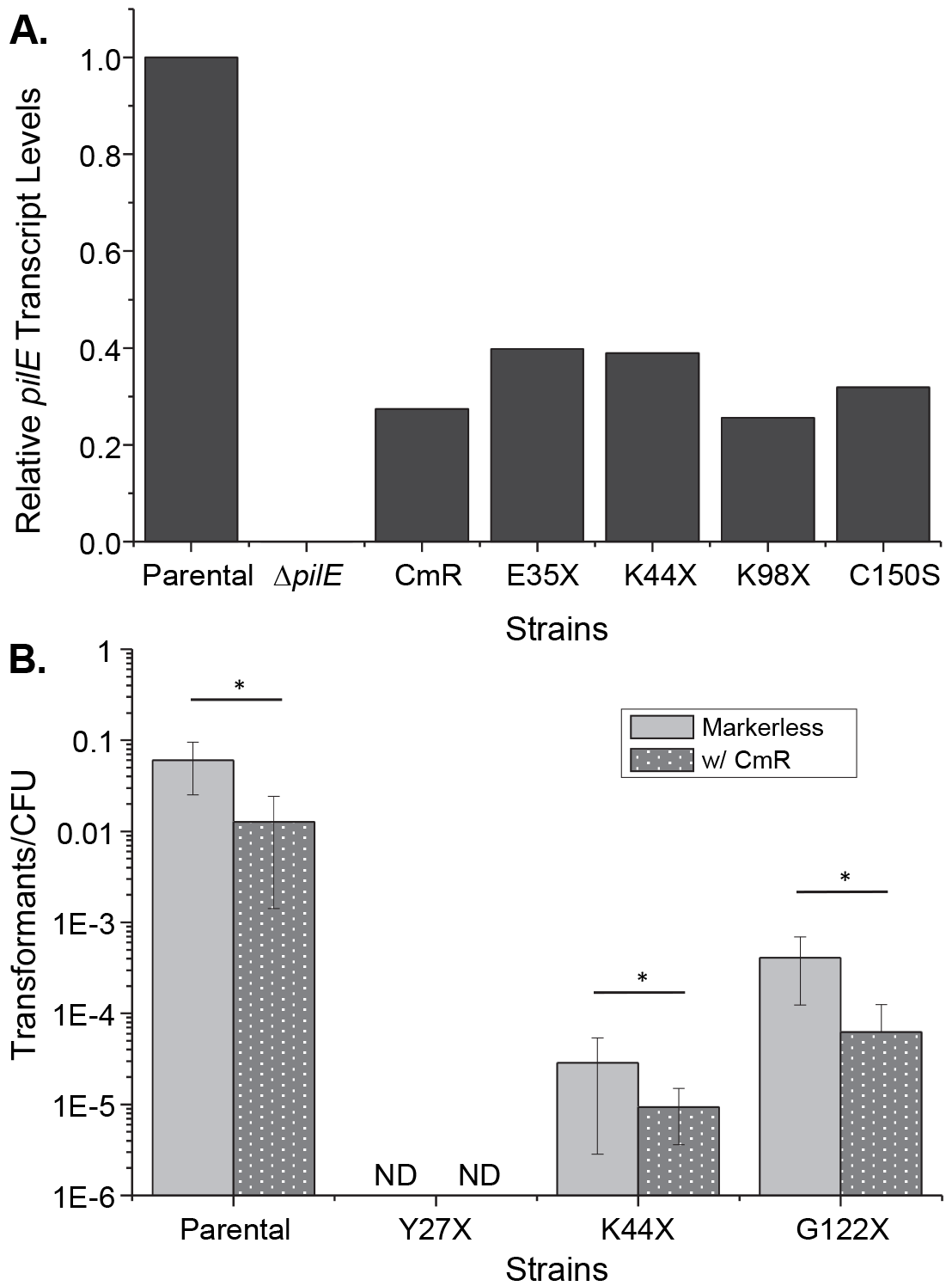

Supplement: S6 Fig — A. Relative mRNA levels of pilE as measured by quantitative RT-PCR. Strains CmR, E35X, K44X, K98X, and C150S contain a CmR. B. Transformation efficiencies of strains either with or without the CmR downstream of pilE. X = nonsense mutation, ND = transformants not detected, *p<0.05 Student’s T-test. (TIF) [file pgen.1006069.s006.tif]
