# Supplementary material for: The Pilin N-terminal Domain Maintains Neisseria gonorrhoeae Transformation Competence during Pilus Phase Variation
Source: PLoS Genet. 2016 May 23;12(5):e1006069. doi: 10.1371/journal.pgen.1006069 (PMC4877100; doi:10.1371/journal.pgen.1006069)
Supplement: S1 Table — alocation of mutation in pilE resulting in a P- colony morphology. bresultant amino acid change. Red shading indicates a nonsense mutation. Yellow shading indicates a cysteine mutant. cTransformation competence relative to the WT strain (++++) and the non-competent ΔpilE strain. dPilin protein levels relative to the WT strain (++) and the ΔpilE strain (-). eS-pilin protein levels relative to the WT strain (++) and the ΔpilE strain (-). fPilus related colony morphology relative to WT (++) and the ΔpilE strain (—). (DOCX) [file pgen.1006069.s007.docx]

| ^a^Mutated Base | ^b^AA Change | ^c^Transformation  Efficiency | ^d^Pilin  Protein | ^e^S-pilin  Protein | ^f^Colony Morphology |
| --- | --- | --- | --- | --- | --- |
| 361 | Gly_114_ to Arg | ++++ | ++ | ++ | - |
| 362 | Gly_114_ to Ala | ++++ | ++ | ++ | + |
| 364 | Ser_114_ to Pro | ++++ | + | ++ | + |
| 365 | Ser_115_ to Stop | ++ | - | - | -- |
| 368 | Val_116_ to Glu | +++ | - | ++ | - |
| 368 | Val_116_ to Gly | ++ | - | ++ | - |
| 370 | Lys_117_ to Stop | + | - | - | - |
| 373 | Trp_118_ to Arg | + | - | + | - |
| 373 | Trp_118_ to Arg | + | - | ++ | - |
| 373 | Trp_118_ to Gly | ++ | - | +++ | - |
| 374 | Trp_118_ to Ser | ++ | - | ++ | - |
| 374 | Trp_118_ to Leu | ++ | - | + | - |
| 375 | Trp_118_ to Stop | + | - | - | - |
| 375 | Trp_118_ to Cys | + | - | +++ | - |
| 375 | Trp_118_ to Cys | ++ | - | ++ | - |
| 376 | Phe_119_ to Val | +++ | - | ++ | - |
| 379 | Cys_120_ to Ser | ++ | - | - | - |
| 379 | Cys_120_ to Arg | + | - | - | - |
| 379 | Cys_120_ to Gly | ++ | - | - | + |
| 380 | Cys_120_ to Ser | ++ | - | - | - |
| 380 | Cys_120_ to Phe | ++ | - | - | - |
| 381 | Cys_120_ to Stop | ++ | - | - | - |
| 381 | Cys_120_ to Trp | ++ | - | - | + |
| 382 | Gly_121_ to Arg | ++ | - | + | - |
| 382 | Gly_121_ to Stop | ++ | - | - | - |
| 383 | Gly_121_ to Glu | ++ | - | + | -- |
| 383 | Gly_121_ to Ala | + | - | + | - |
| 389 | Pro_123_ to Arg | +++ | - | ++ | -- |
| 391 | Val_124_ to Leu | +++ | - | +++ | - |
| 391 | Val_124_ to Phe | ++ | - | ++ | - |
| 392 | Val_124_ to Gly | ++ | - | ++ | - |
| 394 | Thr_125_ to Pro | ++ | - | +++ | - |
| 397 | Arg_126_ to Ser | +++ | - | ++ | - |
| 397 | Arg_126_ to Cys | + | - | ++ | - |
| 398 | Arg_126_ to His | ++ | - | ++ | - |
| 398 | Arg_126_ to Pro | +++ | - | +++ | + |
| 398 | Arg_126_ to Leu | +++ | - | + | - |
| 403 | Gly_128_ to Cys | +++ | + | ++ | + |
| 439 | Ala_140_ to Thr | ++++ | + | ++ | + |
| 440 | Ala_140_ to Asp | +++ | + | ++ | - |
| 442 | Ile_141_ to Leu | +++ | ++ | ++ | - |
| 443 | Ile_141_ to Thr | ++ | - | ++ | - |
| 443 | Ile_141_ to Ser | ++ | - | ++ | - |
| 451 | Lys_144_ to Stop | ++ | - | - | - |
| 455 | His_145_ to Pro | ++ | - | + | - |
| 457 | Leu_146_ to Val | ++ | - | + | -- |
| 458 | Leu_146_ to Pro | ++ | - | + | - |
| 458 | Leu_146_ to Gln | ++ | - | + | - |
| 460 | Pro_147_ to Thr | ++ | - | + | - |
| 460 | Pro_147_ to Ala | +++ | - | ++ | - |
| 460 | Pro_147_ to Ser | +++ | - | ++ | - |
| 461 | Pro_147_ to Leu | ++ | - | + | - |
| 461 | Pro_147_ to Gln | +++ | - | + | -- |
| 461 | Pro_147_ to Arg | ++ | - | - | - |
| 464 | Ser_148_ to Stop | ++ | - | - | - |
| 464 | Ser_148_ to Stop | ++ | - | - | + |
| 467 | Thr_149_ to Asn | ++++ | ++ | ++ | + |
| 469 | Cys_150_ to Ser | ++ | - | - | + |
| 469 | Cys_150_ to Arg | + | - | - | - |
| 469 | Cys_150_ to Gly | ++ | - | - | - |
| 470 | Cys_150_ to Tyr | ++ | - | - | - |
| 470 | Cys_150_ to Ser | ++ | - | - | - |
| 470 | Cys_150_ to Phe | ++ | - | - | - |
| 471 | Cys_150_ to Stop | ++ | - | - | - |
| 471 | Cys_150_ to Trp | ++ | - | - | - |
| 472 | Arg_151_ to Gly | ++ | - | - | - |
| 472 | Arg_151_ to Cys | +++ | + | ++ | - |
| 473 | Arg_151_ to Pro | ++ | - | ++ | + |

^a^location of mutation in *pilE* resulting in a P- colony morphology

^b^resultant amino acid change. Red shading indicates a nonsense mutation. Yellow shading indicates a cysteine mutant.

^c^Transformation competence relative to the WT strain (++++) and the non-competent Δ*pilE* strain

^d^Pilin protein levels relative to the WT strain (++) and the Δ*pilE* strain (-)

^e^S-pilin protein levels relative to the WT strain (++) and the Δ*pilE* strain (-)

^f^Pilus related colony morphology relative to WT (++) and the Δ*pilE* strain (--)
