# Supplementary material for: The Pilin N-terminal Domain Maintains Neisseria gonorrhoeae Transformation Competence during Pilus Phase Variation
Source: PLoS Genet. 2016 May 23;12(5):e1006069. doi: 10.1371/journal.pgen.1006069 (PMC4877100; doi:10.1371/journal.pgen.1006069)
Supplement: S2 Table — (DOCX) [file pgen.1006069.s008.docx]

| **Primer #** | **Primer name** | **Sequence (5’-3’)** |
| --- | --- | --- |
| KP001 | 5' *pilE* 57 | CGTCGGCATTTTGGCGGCAG |
| KP002 | 3' *pilE* 850 | GCGGGCTGGATTCATTTTCGGC |
| KP005 | DUS 5' CAT 1 | GCCGTCTGAAAGAGGTTCCAACTTTCACCG |
| KP006 | CATR1 | CACTTATTCAGGCGTAGCAC |
| KP012 | Crick 5' CAT 1 | TTCAGACGGCATAGAGGTTCCAACTTTCACCA |
| KP013 | *pilE* M361-426 Deg | AAGCGTCAAGACGGTTCGGTAAAATGGTTCTGCGGACAGCCGGTTACGCGCACCGGCGACAACGACGACACCGTTGCCGACGCCAACAAC |
| KP014 | *pilE* M427-492 Deg | GACACCGTTGCCGACGCCAACAACGCCATCGACACCAAGCACCTGCCGTCAACCTGCCGCGATGAATCATCTGCCACCTAAGGCAAATTA |
| KP016 | *pilE* 468 2x Stop HVT | CTGCCGCGATTAATGATCTGCCACC |
| KP021 | *pilE* 355 2x Stop Cys1 | CAAGACGGTTAGGTATAATGGTTCTGCGG |
| KP023 | *pilE* 2x Stop + Cys1 | GACGGTTAGGTATAATGGTTCAGCGGACAG |
| KP024 | *pilE* 374 AA122 Stop | GGTTCTGCGGATAGCCGGTTAC |
| KP026 | *pilE* 366 Cys1 | GGTAAAATGGTTCAGCGGACAGCC |
| KP040 | 5' *pilE* -53 | GATGCCGATGGCGTAAGCCTGAG |
| KP043 | *pilE* AA 31 stop | CCTTCCCGCCTAACAAGACTACACC |
| KP044 | *pilE* AA 52 stop | CGAAGGTCAAAAATGAGCCGTCAC |
| KP045 | *pilE* AA 56 stop | CAGCCGTCACCTAGTATTACCTGAATC |
| KP046 | *pilE* AA 77 stop | GGCATCCGCTTAAACAATCAAAGGC |
| KP047 | *pilE* AA 88 stop | AATATGTTCAGAAAGTTTAAGTCGCAAAAGG |
| KP048 | *pilE* AA 105 stop | CGGCGTAAACTAAGAAATCCAAGAC |
| KP049 | *pilE* AA 34 stop | CTACCAAGACTAAACCGCCCGC |
| KP050 | *pilE* AA 42 stop | GCAAGTTTCCTAAGCCATCCTTTTGG |
| KP051 | *pilE* AA 46 stop | CCGAAGCCATCCTTTAAGCCGAAG |
| KP054 | *pilE* AA 49 stop | CCTTTTGGCCGAATGACAAAAATCAGC |
| KP055 | *pilE* AA 50 stop | CGAAGGTTAAAAATCAGCCGTCACCG |
| KP056 | *pilE* AA 51 stop | CGAAGGTCAATAATCAGCCGTCACC |
| KP155 | S-pil Clvg Mut +122 | CCGAAGCCATCGCTGCAATGGAAGGTCAAAAT C |
| KP157 | +KpnI *comP* 5' -8 | GGGGTACCGTAAAGTAATGACTGATAATCGGGG |
| KP158 | +XhoI *comP* 3' | GGGTCTCGAGCCGCACAAATACTGAAACAC |
| KP159 | (2) *pilD* Clvg Mut +3 | GAATACCCTTCAAAAAAGTTTTACCCTTATCG |
| KP162 | *pilE* AA52 SAV-TMA | GGTCAAAAAACCATGGCCACCGAGTATTAC |
| KP170 | *pilE* RT Set2 5' +33 | CGAGCTGATGATTGTGATCG |
| KP171 | *pilE* RT Set2 3' +136 | AAGGATGGCTTCGGAAACTT |
| KP173 | *pilE* 3' +550 + DUS | ATGCCGTCTGAAGCCGTGGGAAATCACTTACCGTT |
| KP174 | *pilE* 5' -53 + DUS | ATGCCTCTGAAGATGCCGATGGCGTAAGCCTGAG |
| KP176 | *comP*5’ | TGGCGGGACGGCATCTGTA |
| KP177 | *comP*3’ | TCGAACGCGGCCTTTTGTATTT |
| KP178 | *pilV*5’BamHI | GCGCGGATCCACAACGCCGCCTGACGCTTAT |
| KP179 | *pilV*3’EcoRI | GCGCGAATTCTCGCCCGCTACATTTTTATTCA |
| KP180 | *pilV*mutkpnFor | CATCCTGACGCTCATCGGGTACCCATCCTATAAAACCTAC |
| KP181 | *pilV*mutkpnRev | GTAGGTTTTATAGGATGGGTACCCGATGAGCGTCAGGATG |
| KP182 | *omp3*FOR | AGCAGGCTCCTCAATATGTT |
| KP183 | *omp3*REV | CTTGAGTCATTTGCGCTTGA |
| KP221 | 3’ *pilE* +550 | GCCGTGGGAAATCACTTACCGCTT |
| KP222 | 5’ *pilE* +1 | ATGAATACCCTTCAAAAAGGCTTTACCC |
| KP227 | P.a. Spil | GTTTCCGAAGCCTTAGCAAGCGTCAACCCGCTGAAATCAGCCG |
